# Clinical features and survival of multiple myeloma patients harboring t(14;16) in the era of novel agents

**DOI:** 10.1038/s41408-020-0307-4

**Published:** 2020-04-14

**Authors:** Roberto Mina, Nisha S. Joseph, Francesca Gay, Efstathios Kastritis, Maria Teresa Petrucci, Jonathan L. Kaufman, Vittorio Montefusco, Maria Gavriatopoulou, Francesca Patriarca, Paola Omedé, Lawrence H. Boise, Maria Roussou, Nicola Giuliani, Stefania Oliva, Massimo Offidani, Angelo Belotti, David L. Jaye, Lorenzo De Paoli, Evangelos Terpos, Sagar Lonial, Mario Boccadoro, Ajay K. Nooka, Meletios A. Dimopoulos

**Affiliations:** 1 Myeloma Unit, Division of Hematology, University of Torino, Azienda Ospedaliero-Universitaria Città della Salute e della Scienza di Torino, Torino, Italy; 2 Hematology and Medical Oncology, Winship Cancer Institute, Emory University, Atlanta, GA 30322 USA; 3 Department of Clinical Therapeutics, National and Kapodistrian University of Athens, School of Medicine, Athens, Greece; 4 Hematology, Azienda Policlinico Umberto I, Sapienza University of Rome, Rome, Italy; 5 Hematology Department, Fondazione IRCCS Istituto Nazionale dei Tumori, Milano, Italy; 6 Clinica Ematologica, Azienda Sanitaria Universitaria Integrata, DAME, Università di Udine, Udine, Italy; 7 Dipartimento di Medicina e Chirurgia, Università di Parma, Parma, Italy; 8 Clinica di Ematologia, Azienda Ospedaliero-Universitaria Ospedali Riuniti di Ancona, Ancona, Italy; 9 Division of Hematology, Spedali Civili di Brescia, Brescia, Italy; 10 Department of Pathology and Laboratory Medicine, Emory University, Atlanta, GA 30322 USA; 11 Division of Hematology, Department of Translational Medicine, University of Eastern Piedmont, Novara, Italy

**Keywords:** Myeloma, Medical research

Dear Editor,

Multiple myeloma (MM) is a clonal plasma cell disorder whose prognosis is driven by the presence or absence of a wide gamut of primary (trisomies and translocations) and secondary (monosomies/deletions and amplifications) genetic abnormalities. Translocations involving the immunoglobulin heavy chain (IgH) region on chromosome 14 and one of its partners on chromosomes 4, 6, 11, 16 and 20 occur in approximately 40% of MM patients. Among the IgH translocations, t(11;14) is the most commonly observed lesion (occurring in 16–24% of MM patients), while t(14;16) is rather uncommon (<5% of patients, in most reported series)^1–3^. The International Myeloma Working Group (IMWG)^4,5^ and the m-SMART^6^ risk-stratification currently consider t(11;14) as a standard-risk chromosomal abnormality^7^, hile t(4;14), t(14;16) and t(14;20) have been associated with poor survival, although the data on the prognostic impact of t(14;16) in the era of newer anti-myeloma drugs have not been extensively reported and few retrospective studies have reported conflicting results on the prognostic significance of this abnormality^1,8,9^. t(14;16)(q32;q23) involves the IgH locus and the c-musculoaponeurotic fibrosarcoma (c-MAF) oncogene locus, the latter likely being responsible, at least partially, of the MAF-mediated innate resistance to proteasome inhibition, a backbone treatment in current myeloma regimens^10^.

Having considered the availability of newer combination regimens and the recent outcomes in the treatment of MM, we conducted a multi-institutional collaborative study among patients harboring t(14;16) diagnosed at different European and American centers, with the aim of describing their clinical features and investigating the prognostic value of t(14;16).

Newly diagnosed (ND)MM patients meeting the IMWG criteria for symptomatic MM were included in the analysis. Patients were diagnosed between December 2006 and March 2017^11,12^ and registered in databases of: clinical trials coordinated by the Italian group and with centralized fluorescent in situ hybridization (FISH) at the Torino site (TO, Italy; see Table S1 for the list of source trials with centralized FISH at TO); the Winship Cancer Institute (WCI, Emory University, Atlanta US); and the Department of Clinical Therapeutics, National and Kapodistrian University of Athens (NKUA, Greece). Data regarding demographic and baseline characteristics, treatments administered, responses to treatment and survival outcomes were collected from the institutional review board databases approved by the respective centers. Response to treatment and disease progression were assessed using the IMWG uniform response criteria^13,14^.

FISH analysis was performed on CD138+ enriched bone marrow plasma cells; the cut-off levels ranged from 10 to 20% for numerical aberrations and from 5 to 15% for IgH translocations.

Progression-free survival (PFS) was calculated from the date of initial therapy to either the date of the first relapse or death due to any cause; overall survival (OS) was calculated from the date of initial therapy to the date of death. PFS and OS were estimated using the Kaplan–Meier method and compared between groups using the log-rank test. We used the Cox proportional hazards model to identify factors affecting PFS and OS. A two-tailed *P*-value < 0.05 was considered significant for all statistical tests. Data were analyzed as of April 2018 using R (v3.1.1).

We identified 123 NDMM patients with t(14;16)-positive FISH at diagnosis (TO, *n* = 76/1678; WCI, *n* = 29/926; NKUA, *n* = 18/571) with a median age at diagnosis of 66 years (range, 38–87 years; see Table S2. *Patient characteristics at diagnosis*). Twenty-three percent of patients had lactate dehydrogenase (LDH) levels above the upper limit of normal; creatinine ≥2 mg/dl and hypercalcemia were observed in 16% of patients. Most of the patients presented with International Staging System (ISS) stages 2 (34%) or 3 (43%) and with concomitant chromosomal abnormalities, including del(13q) (71%), gain(1q) (51%), and del(17p) (23%).

All but one patient received novel agents as part of induction therapy, including immunomodulatory drugs (42%), proteasome inhibitors (29%) or a combination of both (28%) (Table S2). Autologous stem-cell transplantation (ASCT) was performed in 42% of patients, 28% upfront and 14% at relapse; 41% of patients received maintenance therapy.

The objective response rate to first-line treatment was 85% (partial response [PR]: 26%; very-good PR [VGPR]: 29%; at least complete response [≥CR]: 28%); 5% of patients were primary refractory to induction therapy.

After a median follow-up of 53 months (95% CI 35–63), median PFS and OS for the entire cohort were, respectively, 19 months (95% CI 16–30) and 53 months (95% CI 36–63), with 39% of patients being alive at 5 years.

t(14;16)-positive patients with del(17p), del(13q), or amp(1q) (*n* = 101) had significantly shorter median PFS (17 months vs. NR; HR: 3.33, *p* = 0.04) and inferior, although not statistically significant, OS (median, 46 months vs. NR; HR: 1.54, *p* = 0.47), as compared to those without additional chromosomal abnormalities (*n* = 10) (Fig. 1). More specifically, the risk of progression or death of t(14;16) patients increased by 1.35 fold in patients also harboring del(17p) (*p* = 0.23), by 1.64 in presence of del(13q) (*p* = 0.05) and by 2.2 in patients carrying amp(1q) (*p* = 0.02); a shorter OS, although not statistically significant, was observed in presence of del(17p) (HR: 1.3; *p* = 0.47) and amp(1q) (HR: 1.7; *p* = 0.16), whereas a significantly shorter OS was observed with del(13q) (HR: 1.96; *p* = 0.04).

**Fig. 1:**
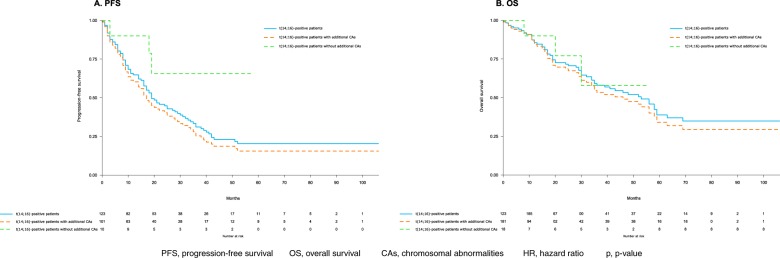
Time-to-event analysis. Kaplan–Meier progression-free survival (**a** *PFS* progression-free survival) and overall survival (**b** *OS* overall survival) curves in t(14;16)-positive patients. Blue curve: t(14;16)-positive patients overall; orange curve: t(14;16)-positive patients with additional *CAs* chromosomal abnormalities [del(17p) and/or del(13q) and/or amp(1q)]; green curve: t(14;16)-positive patients without additional CAs.

Among younger, ASCT-eligible patients, median PFS and OS were, respectively, 24 (95% CI 17–38) and 52 months (95% CI 34–69); ASCT upfront prolonged median PFS (36 vs. 17 months; HR: 0.5, *p* = 0.036), as compared to standard-dose chemotherapy without ASCT. Maintenance therapy significantly prolonged median PFS, as compared to no maintenance (36 vs. 19 months, HR: 0.59; *p* = 0.045). The achievement of ≥CR as best response to treatment was related to prolonged PFS (HR 0.29, *p* < 0.001), as compared to the achievement of PR/VGPR.

In a multivariate analysis, ISS-3 (HR: 1.79; *p* = 0.014) and the presence of an additional chromosomal abnormality (del(17p), del(13q) or amp(1q); HR: 3.24; *p* = 0.049) were associated to a shorter PFS, while ISS-3 (HR: 1.6; *p* = 0.09), hypercalcemia (HR: 2.4; *p* = 0.043) and elevated LDH (HR: 2.13; *p* = 0.026) were associated with a significantly shorter OS (see Table S3*. Univariate and multivariate analyses*).

To our knowledge, this is one of the largest studies on NDMM patients harboring t(14;16) describing their clinical features and survival outcomes in the era of novel agents.

In a previous report from the Mayo Clinic (2003), the presence of t(14;16) was associated with shorter PFS (median, 9 vs. 30 months) and OS (median, 16 vs. 41 months), as compared to t(14;16)-negative MM (see Table S4. *Previous reports of t(14;16)*)^1^. However, that analysis included a limited number of patients (*n* = 15) before the introduction of novel agents. These results were not confirmed in a larger cohort of patients (*n* = 1003) treated by the Intergroupe Francophone du Myélome (IFM): here, t(14;16) was detected in 32 patients and was not prognostically significant^9^. Nonetheless, the IMWG listed t(14;16) among the unfavorable high-risk chromosomal abnormalities^7^. Narita et al. showed that both PFS (median, 0.6 vs. 1.2 years) and OS (median, 3.06 vs. 4.40 years) were significantly shorter among t(14;16)-positive patients (*N* = 35) than among t(14;16)-negative patients (*N* = 124)^8^.

According to our analysis, the majority of t(14;16) patients presented at diagnosis with at least one other high-risk feature, such as additional chromosomal abnormality (81%), ISS-3 (43%) and elevated LDH (23%), which were all significantly associated with inferior survival (Table S3).

Median PFS and OS for the entire cohort were 19 and 53 months. Although this study does not include a control population, the median OS of t(14;16) patients was shorter than that observed in a cohort of patients treated with novel agents (median, 72 months)^15^. Interestingly, t(14;16)-positive patients who harbored additional chromosomal abnormalities [del(17p), del(13q), or amp(1q)] displayed worse PFS (HR: 3.33) and OS (HR: 1.54), as compared to t(14;16) patients without further chromosomal abnormalities (Table S3). Despite the limited number of patients, this observation casts doubt on the unfavorable prognostic significance of isolated t(14;16), which seems to occur infrequently, and raises the question of whether the poor prognosis of these patients is related to t(14;16) per se rather than to the presence of additional genetic lesions.

Our analysis also confirmed the role of upfront ASCT in prolonging PFS and OS and the role of maintenance treatment in deepening the quality of response and prolonging PFS as compared to fixed-duration therapy.

This study has some limitations. First, the absence of a control population limits our ability to precisely estimate the risk conferred by the presence of t(14;16) in the era of novel agents. Secondly, the heterogeneity of treatment therapies does not allow us to speculate on the efficacy of specific regimens. Finally, since 62% of patients in this cohort were enrolled in clinical trials, the rates of patients with renal failure or aggressive disease requiring immediate treatment were underestimated and consequently affected the results. Despite these caveats, PFS and OS of t(14;16) patients in the era of novel agents seem to be shorter than those of standard-risk patients^15^. Whether the poor prognosis of t(14;16) patients is associated with t(14;16) per se or with the frequent co-existence of other high-risk features is an issue that needs to be addressed.

## Supplementary information

Supplementary Appendix
