# Supplementary material for: Clinical features and survival of multiple myeloma patients harboring t(14;16) in the era of novel agents
Source: Blood Cancer J. 2020 Apr 14;10(4):40. doi: 10.1038/s41408-020-0307-4 (PMC7156404; doi:10.1038/s41408-020-0307-4)
Supplement: Supplementary file 1 — Supplementary Appendix [file 41408_2020_307_MOESM1_ESM.pdf]

Clinical Features and Survival of Multiple Myeloma Patients Harboring t(14;16) in the Era of Novel Agents

Supplementary Appendix

Summary

- **Table S1.** List of the source trials with centralized FISH at the Torino Site
- **Table S2.** Patient characteristics at diagnosis
- **Table S3.** Univariate and multivariate analyses
- **Table S4.** Previous reports of t(14;16)

Table S1. List of the source trials with centralized FISH at the Torino Site

Trial		Total N of patients
**IST-CAR-506**[1]	ClinicalTrials.gov ID: NCT01346787	51
**IST-CAR-561**[2]	ClinicalTrials.gov ID: NCT01857115	43
**IST-CAR-601**[3]	ClinicalTrials.gov ID: NCT02204241	29
**EMN01**[4]	ClinicalTrials.gov ID: NCT01093196	486
**RV-MM-EMN-441**[5]	ClinicalTrials.gov ID: NCT01091831	250
**RV-MM-PI-209**[6]	ClinicalTrials.gov ID: NCT00551928	243
**GIMEMA-MM-04-05** *and* **GIMEMA-MM-05-05**[7,8]	EudraCT Numbers: 2005-004714-32 2005-004730-41	72
**GIMEMA-MM-03-05**[9,10]	ClinicalTrials.gov ID: NCT01063179	376
**26866138MMY2069**[11]	ClinicalTrials.gov ID: NCT01190787	128

FISH, fluorescence in situ hybridization.

Table S2. Patient characteristics at diagnosis

Characteristics	Patients n=123
**Age**, median (range)	66 (38-87)
**Sex**, male	57 (46%)
**Hemoglobin** (g/dl), median, range	10 (5.2-15.6)
**Platelets** <100,000 x 10^9/mm3	13 (14%)
Serum **calcium** > ULN	15 (16%)
Serum **creatinine** ≥ 2 mg/dl	19 (16%)
**Bone marrow plasma cells** %, median (range)	60 (0-100)
**M-spike** (g/dl), median (range)	3.3 (0-11.6)
**Lactate dehydrogenase** > ULN	23 (23%)
M-protein **isotype**	
IgG	67 (54%)
IgA	32 (26%)
Light-chain only	23 (20%)
Missing	1 (<1%)
**ISS**	
1	25 (20%)
2	41 (34%)
3	53 (43%)
Missing	4 (3%)
**FISH**	
del(13q)	87 (71%)
amp(1q)	33 (51%)
del(17p)	27 (23%)
t(4;14)	14 (11%)
t(11;14)	6 (6%)
**Induction treatment**	
IMiD-based (thalidomide or lenalidomide)	52 (42%)
PI-based (bortezomib)	36 (29%)
IMiD plus PI-based	34 (28%)
**ASCT**	52 (42%)
Upfront	34 (69%)
At relapse	18 (31%)
**Maintenance**	50 (41%)
IMiD	28 (56%)
PI	7 (14%)
IMiD + PI	15 (30%)

% are calculated on the number of available data.
ULN, upper limit of normal; M, monoclonal; ISS, International Staging System; FISH, fluorescent in situ hybridization; t, translocation; del, deletion; amp, amplification; t, translocation; IMiD, immunomodulatory drug; PI, proteasome inhibitor; ASCT, autologous stem-cell transplantation.

Table S3. Univariate and multivariate analyses

	UNIVARIATE ANALYSIS				MULTIVARIATE ANALYSIS			
	PFS		OS		PFS		OS	
	HR	p	HR	p	HR	p	HR	p
**ISS 1-2 *vs.* 3**	2	0.002	2.2	0.003	1.86	0.014	1.6	0.09
**LDH <ULN *vs.* >ULN**	1.9	0.017	3.1	<0.001	1.52	0.14	2.15	0.026
**Calcium <ULN *vs.* >ULN**	2.5	0.008	4.8	<0.001	1.34	0.42	2.4	0.043
**+ del(17p) *or* del(13q) *or* amp(1q)**	3.35	0.04	1.6	0.46	3.24	0.049	1.3	0.66

ISS, International Staging System stage; LDH, lactate dehydrogenase; ULN, upper limit of normal; del, deletion; amp, amplification; t, translocation; PFS, progression-free survival; OS, overall survival; HR, hazard ratio; p, p-value.

Table S4. Previous reports of t(14;16)

**Author, year** [Reference]	**N of patients**	**Median PFS, months (range)**	**Median OS, months (range)**
Mina et al., 2020	123	19 (16-30)	53 (36-63)
R. Fonseca et al., 2003[12]	15	9 (13-22)	30 (27-32)
H. Avet-Loiseau et al., 2011[13]	32	HR: 1.28, p=NS	HR: 1.25, p=NS
T. Narita et al., 2015[14]	35	7	37
A. Jurczyszyn et al., 2018[15]	213	31 (28-40)	88 (49-177)

N, number; PFS, progression-free survival; OS, overall survival; rng, range; HR, hazard ratio; p, p-value; NS, not significant.

Additional references

1 Bringhen S *et al.* Carfilzomib, cyclophosphamide, and dexamethasone in patients with newly diagnosed multiple myeloma: A multicenter, phase 2 study. *Blood* 2014; 124: 63–69.

2 Bringhen S *et al.* Phase 1/2 study of weekly carfilzomib, cyclophosphamide, dexamethasone in newly diagnosed transplant-ineligible myeloma. *Leukemia* 2018; 32: 979–985.

3 Bringhen S *et al.* Carfilzomib, Cyclophosphamide and Dexamethasone (KCyd) in Elderly Newly Diagnosed Multiple Myeloma (NDMM) Patients: Initial Results of a Phase 1 Study. *Haematologica* 2016; 101: 520 [Abstract #E1260, EHA 2016 21st Congress].

4 Magarotto V *et al.* Triplet vs doublet lenalidomide-containing regimens for the treatment of elderly patients with newly diagnosed multiple myeloma. *Blood* 2016; 127: 1102–8.

5 Gay F *et al.* Chemotherapy plus lenalidomide versus autologous transplantation, followed by lenalidomide plus prednisone versus lenalidomide maintenance, in patients with multiple myeloma: a randomised, multicentre, phase 3 trial. *Lancet Oncol* 2015; 16: 1617–1629.

6 Palumbo A *et al.* Autologous transplantation and maintenance therapy in multiple myeloma. *N Engl J Med* 2014; 371: 895–905.

7 Palumbo A *et al.* Bortezomib as induction before autologous transplantation, followed by lenalidomide as consolidation-maintenance in untreated multiple myeloma patients. *J Clin Oncol* 2010; 28: 800–807.
8 Gay F *et al.* Bortezomib induction, reduced-intensity transplantation, and lenalidomide consolidation-maintenance for myeloma: updated results. *Blood* 2013; 122: 1376–83.
9 Palumbo A *et al.* Bortezomib-melphalan-prednisone-thalidomide followed by maintenance with bortezomib-thalidomide compared with bortezomib-melphalan-prednisone for initial treatment of multiple myeloma: A randomized controlled trial. *J Clin Oncol* 2010; 28: 5101–5109.
10 Palumbo A *et al.* Bortezomib-Melphalan-Prednisone-Thalidomide Followed by Maintenance With Bortezomib-Thalidomide Compared With Bortezomib-Melphalan-Prednisone for Initial Treatment of Multiple Myeloma: Updated Follow-Up and Improved Survival. *J Clin Oncol* 2014; 32: 634–640.
11 Larocca A *et al.* A phase 2 study of three low-dose intensity subcutaneous bortezomib regimens in elderly frail patients with untreated multiple myeloma. *Leukemia* 2016; 30: 1320–1326.
12 Fonseca R *et al.* Clinical and biologic implications of recurrent genomic aberrations in myeloma. *Blood* 2003; 101: 4569–4575.
13 Avet-Loiseau H *et al.* Translocation t(14;16) and multiple myeloma: is it really an independent prognostic factor? *Blood* 2011; 117: 2009–11.
14 Narita T *et al.* t(14;16)-positive multiple myeloma shows negativity for CD56 expression and unfavorable outcome even in the era of novel drugs. *Blood Cancer J* 2015; 5: e285–e285.
15 Jurczyszyn A *et al.* The Prognostic Impact of t(14;16) in Multiple Myeloma: A Multicenter Retrospective Study of 213 Patients. Is It Time to Revise the Revised ISS? *Blood* 2018; 132: Abstract #4452 [ASH 2018 60th Annual Meeting].
